# Mitogenomes Reveal Novel Phylogeographic Patterns and Temporal Evolutionary History of Barn Swallows and Close Relatives

**DOI:** 10.3390/ijms27167433

**Published:** 2026-08-20

**Authors:** Gianluca Lombardo, Guido Roberto Gallo, Andrea De Benedictis, Marta Cavallini, Giulio Formenti, Giorgio Binelli

**Affiliations:** 1 Department of Biosciences, University of Milan, 20133 Milano, Italy; guido.gallo@unimi.it; 2 Department of Biotechnology and Life Sciences (BDSV), University of Insubria, 21100 Varese, Italygiorgio.binelli@uninsubria.it (G.B.); 3 The Vertebrate Genome Laboratory, The Rockefeller University, York Ave 1230, New York, NY 10021, USA

**Keywords:** barn swallow, *Hirundo rustica*, mitogenome haplogroups, mitogenome phylogeography, *Hirundo rustica tytleri*, swallow phylogeography, evolutionary history

## Abstract

We investigated mitochondrial diversity and phylogeographic structure across all subspecies of the barn swallow (*Hirundo rustica*) to clarify evolutionary relationships, identify novel haplogroups, and assess the extent of hybridisation and maternal introgression. We analysed 580 complete *H. rustica* mitochondrial genomes together with twelve additional *Hirundo* species, three of which were newly obtained. We identified 553 unique haplotypes, including 166 previously undescribed ones, revealing high nucleotide diversity among subspecies. Phylogenetic analyses found five major haplogroups. Of these, a novel haplogroup (E) was discovered and ascribed to *H.r. tytleri*, previously considered part of *H.r. erythrogaster*. Comparative analyses with other *Hirundo* species support a southern African origin of *H. rustica* at the end of the Pliocene, followed by diversification and northward expansion. Our comprehensive dataset substantially refines the phylogeographic resolution of the species. We identify a novel haplogroup, clarify subspecies-level relationships, and reveal pervasive hybridisation and maternal introgression. These findings revise the evolutionary history of *H. rustica* and underscore the complexity of its population structure and gene flow. Beyond reconstructing lineage history, our study provides an evolutionary framework crucial for predicting environmental change responses and guiding migratory species conservation strategies.

## 1. Introduction

The iconic barn swallow (*Hirundo rustica*) stands out of the flocks as one of the most extensively distributed avian species in the world [1]. This phenomenon is mostly attributed to its social behaviour regarding nesting, which in many populations has switched from natural nesting sites like caves and cliffs to human-made structures [2]. The species is commensal with humans and its history is intertwined with our culture as well. Swallows appear in art, poetry, legends and myths of different cultures [3]. The *H. rustica* complex comprises at least five subspecies: *Hirundo rustica rustica* (*H.r. rustica*), breeding in Eurasia and North Africa; *H.r. savignii*, a non-migratory resident of Egypt; *H.r. gutturalis*, breeding in Japan, Korea, China, Mongolia and Eastern Russia; *H.r. tytleri*, breeding in Russia on the border with Mongolia and China; and *H.r. erythrogaster*, breeding in the northern hemisphere from Alaska to Mexico. Another population has been historically defined in Israel as the resident *H.r. transitiva* subspecies [4]. Taxonomy of the latter has been based traditionally on both morphology (particularly plumage colour, breastbands, size and tail lengths) and behaviour, given its non-migratory status. However, recent studies have shown that the genetic difference in mitochondrial DNA (mtDNA) within *H.r. transitiva* and between *rustica* are of the same magnitude, indicating they are the same subspecies [5,6], at least with respect to their maternal lineages. Moreover, hybrid zones have been described mainly using SNPs and with only limited evidence from mtDNA, and they involve all subspecies except for the American *H.r. erythrogaster,* which appears isolated in the Americas. In the Levant region, the migratory routes of *rustica* overlap with those of *H.r. savignii* and *H.r. transitiva*, leading to genetic admixture such as *H.r. savignii* mitochondrial DNA found in phenotypical *H.r. transitiva* samples and *rustica* mitogenomes found in *H.r. savignii* samples and *H.r. transitiva* [5,6,7,8]. On the other hand, in the East, migratory divides exist between *rustica* and *tytleri* in the Siberian Oblast of Irkutsk; *rustica* and *gutturalis* in the Qinghai and Gansu regions of China; and *tytleri* and *gutturalis* in NE Mongolia and Buryatia-Zabaykalsk in Russia [6,9,10,11].

Advances in genomic technologies, particularly whole genome sequencing (WGS), shotgun sequencing, and genotyping-by-sequencing, have dramatically increased the amount and the availability of genetic data. However, most mitochondrial reads from WGS datasets remain underutilised, while harbouring great potential for phylogenetic analyses, especially in unmapped, and usually neglected, mitochondrial reads. To date, genomic studies of barn swallows have primarily focused on nuclear genome structure [12], including the first species pangenome [13], and on population history and admixture [14]. These efforts enabled a deeper exploration of the conservation and evolutionary dynamics within the barn swallow genome, especially for *H.r. rustica*, *H.r. savignii* and *H.r. erythrogaster,* leading to the identification of a comprehensive set of genetic markers and candidate genomic regions under selection.

Both mitochondrial and nuclear data support the monophyly of the species and identify two major phylogenetic clusters, these being 1. *H.r. rustica* (and *H.r. transitiva*) with *H.r. savignii* and 2. *H.r. gutturalis* with *H.r. erythrogaster* and *H.r. tytleri* [2,5,6,7,14,15,16]. The origin of an ancestral barn swallow was dated to ~277 thousand years ago (kya) [6]. Despite ongoing sequencing efforts, data on *H.r. gutturalis* and *H.r. tytleri* subspecies remain minimal, particularly regarding complete mitochondrial genomes, resulting in imprecise divergence time estimates. Here, we explicitly test evolutionary hypotheses about the origins and diversification of *H. rustica*. Specifically, we ask whether:

- Distinct mitochondrial haplogroups correspond to postglacial expansions;
- Mitochondrial relationships amongst subspecies reflect recent secondary contact or long-term incomplete lineage sorting (ILS);
- Where the ancestral mitochondrial lineage of *H. rustica* originated and which historical dispersal pathways gave rise to its modern distribution.

By posing these questions, our study applies mitogenomic tools to understand how historical processes shape current genetic diversity and adaptive potential in a model migratory bird.

## 2. Results

### 2.1. Subspecies Genetic Diversity in Barn Swallows

In silico extraction of mitochondrial reads was employed to retrieve 168 novel *Hirundo rustica* complete mitogenomes belonging to all postulated subspecies spanning the entire breeding range of the species. Sequences taken from GenBank were all from whole genome studies and were therefore not enriched for mitochondrial reads. After duplicate and adapter removal, the number of average reads per sample was ~8000, which was roughly 15% of the total genomic reads. These were enough to cover, on average, 93% of the entire mtDNA sequence with a mean coverage of 30X. Average GC fraction was 44.9%. Coding regions (nps: 1–14860; 16068–16741; 18075–18143) (Figure S1) of new barn swallow samples revealed 166 new haplotypes (Haplotype diversity, Hd = 0.99). When considering the complete dataset, a total of 553 unique haplotypes were found (Hd = 0.95) with 1956 polymorphic sites in the coding region and 2039 different mutations, 954 of which were private mutations. Average coding region (15,601 bp) nucleotide diversity (π) for all *Hirundo rustica* individuals is 0.543% (±0.023%). The largest variability was within the *erythrogaster* subspecies, π = 0.195% (±0.024%), and the lowest was in *H.r. savignii,* π = 0.076% (±0.008%) (Table 1, and in detail in Table S1). To check whether differences in size among species had an effect on diversity estimates, the analysis was also performed on 40 randomly selected *H.r. rustica* samples representing the main haplogroups in the correct proportions. The resulting diversity matrix does not differ from that obtained from the original data (Table S2).

### 2.2. Hirundo rustica tytleri, a New Haplogroup in the Barn Swallow Phylogeny

**Figure 1:**
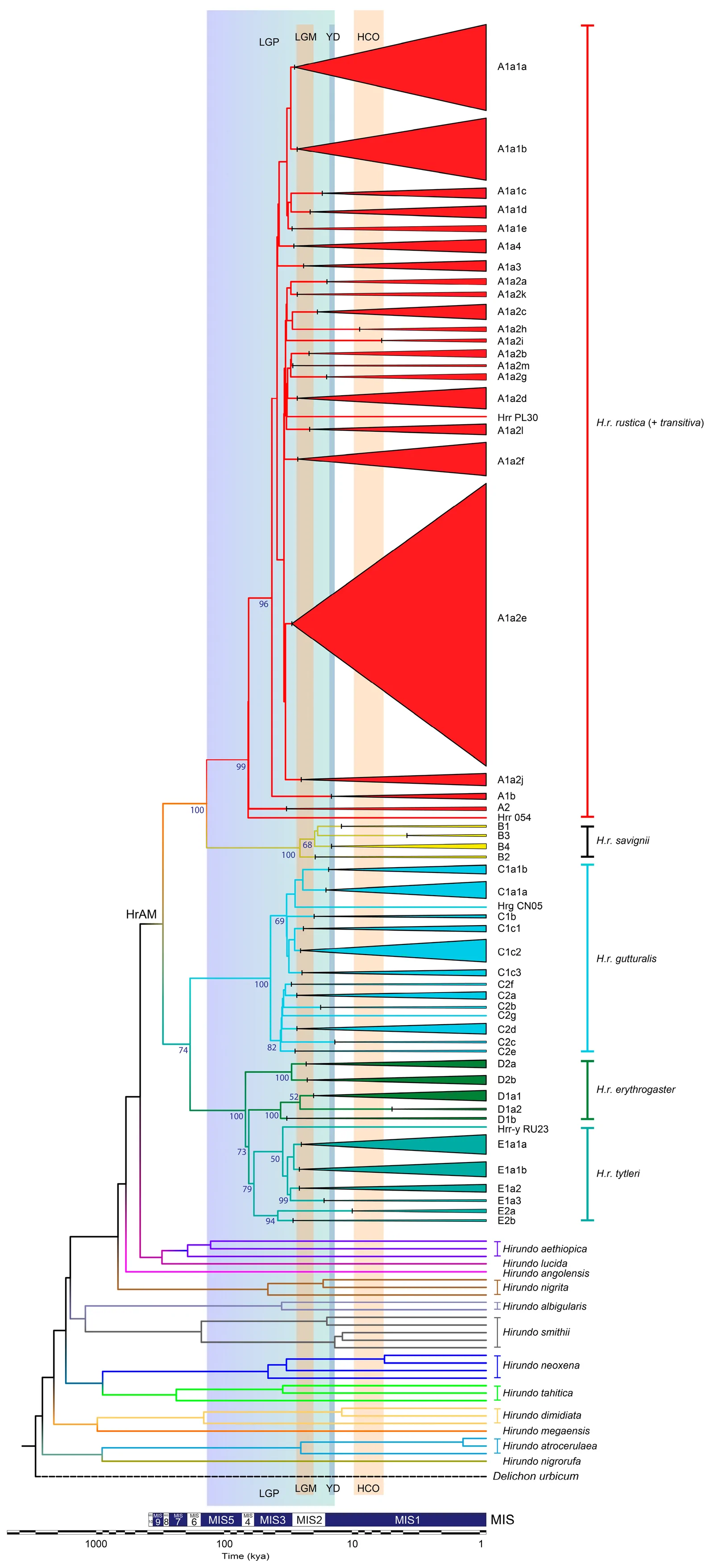
Schematic maximum parsimony phylogeny of *Hirundo rustica* mitogenomes and closest species. This tree was built using the entire mitogenome coding region of 580 barn swallows and 30 other individuals from the genus *Hirundo*. The tree was rooted using *Delichon urbicum* (NC_050298). Major sub-haplogroups of *H. rustica* are represented as triangles which are proportional to the amount of mtDNA present. HrAM refers to the *Hirundo rustica* Ancestral Mitogenome. Colours were assigned based on subspecies for the barn swallow while the other colours indicate the other species. Bootstrap values (1000 iterations) are shown for major haplogroups of *H. rustica*. The timeline (log_10_) at the bottom refers to the Bayesian coalescence times of Supplementary Table S3. Coloured bands indicate the Last Glacial Period (LGP), Last Glacial Maximum (LGM), Younger Dryas (YD), and Holocene Climactic Optimum (HCO); MIS indicates the first 10 Marine Isotope Stages.

Using Bayesian methods for molecular dating, we obtained coalescence times for main divergence events for all five main haplogroups. These are consistent with the mitochondrial lineages previously recovered using *CYB*, *ND2* and complete mtDNA [2,5,6]. Haplogroup A (*N* = 389) expanded in Eurasia around 64 ± 0.7 kya (Table S3). The four main haplogroups of A1a1–A1a4 (Figure S2) all diverged at the beginning of the Last Glacial Period 23–33 ± 0.1 kya (Figure 1). Haplogroup B (*N* = 12) dates to 24 ± 0.3 kya and is subdivided into three main sub-haplogroups, B1 in Israel (already previously described [6]) and B2 and B3 in Egypt (Figure S3). These diverged, respectively, 11, 19 and 3.6 (±0.1) kya. Haplogroup C originated 40 ± 0.3 kya and split into two main haplogroups (Figure S4), C1 and C2, both encompassing samples from Russia, China, Mongolia and Japan indistinctively. Haplogroup C1 dates back to 32 ± 0.2 kya and further differentiates into three sub-haplogroups, C1a-c, once again without geo-specificity (20–30 ± 0.2 kya). Haplogroup C2 originated 35 ± 0.4 kya and splits into five sub-haplogroups: C2a-C2e, C2b and C2e are Mongolia (Ugii Lake area) and China-specific (E-SE China), respectively, all dating to 13–27 ± ~0.2 kya. Other interesting geo-specific sub-haplogroups are found closer to the leaves of the phylogenetic tree; these are C1c1a, Northern/Eastern China, and C2d1a1, circumscribing the Baikal Lake (Southern Siberia) and dated to17 ± 0.1 kya. Haplogroup D (*N* = 24), now dated to 66 ± 0.7 kya in this work (Table S3), is characterised by three main clades (Figure S5), D1, D2 and E. More details on the latter haplogroup are reported later. Both the D1 and D2 haplogroups are also older than previously thought: D1, 35 ± 0.7 kya, and D2, 30 ± 0.2 kya. Th main sub-haplogroups are now D1a (USA and Canada), D2a (Nebraska and Colorado) and D2b (Nebraska) (20–24 ± 0.2 kya). The new haplogroup E diverged from the lineage leading to the American haplogroups D1 and D2 around 58 ± 0.6 kya and split into two main sub-haplogroups (Figure S6), E1 (33 ± 0.2 kya) and E2 (37 ± 0.5 kya), both found in Mongolian and Russian birds. Notably, a Chinese bird registered as a *rustica-gutturalis* hybrid is present in sub-haplogroup E1a1b and an American one in E2a.

### 2.3. The Complete Phylogeographical History of Swallows

We estimated the ancestral barn swallow mitogenome progenitor to have lived 302 ± 2 kya, earlier than previously thought (277 + 24 kya, [6]). From this ancestral population, two main clades diverged. The oldest led to the eastern and American populations (CDE) and diverged 167 ± 3 kya. Then, the ancestor leading to Eurasian and Egyptian subspecies (AB) diverged 128 ± 2 kya. Node AB further splits into haplogroup A (comprising both *H.r. rustica* and *H.r. transitiva*) 64 ± 0.7 kya and haplogroup B 24 ± 0.3 kya. Both dates are older than previously calculated.

We obtained 13 additional mitogenome coding regions belonging to twelve different *Hirundo* species. Their phylogeny roughly follows a south-to-north pattern. Three of these have never been published before (*H. nigrorufa*, *H. megaensis* and *H. lucida*). We found that *H. aethiopica* and *H. lucida* are sister groups still present in the putative origin area of HrAM and are in fact at the base of the *Hirundo rustica* monophyletic clade which separated 302 ± 8 kya. Modern *H. aethiopica* haplogroups are dated to 190 ± 6 kya. *H. angolensis* is at the base of the aforementioned groups, unlike previously seen [7,15], and split around 580 ± 19 kya, followed by *H. nigrita*, which split earlier (661 ± 20 kya) and expanded west, with current mitogenomes having a 48 ± 2 kya common origin. *H. albigularis* and *H. smithii* form a monophyletic sister clade, which split from the main phylogeny in southern Africa around 1462 ± 60 kya; the two species then diversified from one another around 1197 ± 31 kya, with *albigularis* remaining in southern Africa (34 ± 1 kya) while *H. smithii* spread throughout the continent in two major haplogroups (SMI-A, 14 ± 0.2 kya, and SMI-B, 13 ± 0.1 kya). A major split occurred 1617 ± 53 kya between African swallows and the Pacific clade including *H. tahitica* and *H. neoxena*. These diverged from each other 897 ± 55 kya and reached the Australasian and Oriental areas with current ancestries originating, respectively, 225 ± 16 kya and 44 ± 0.8 kya. The second-to-last basal phylogenetic split occurred in Southeast Africa 2039 ± 70 kya and gave rise to the monophyletic sister clade comprising *H. dimidiata* and *H. megaensis*, which had been previously described as the “Pearl-breasted clade”. These two diverged 971 ± 54 kya, with the latter reaching the Oromian region of Ethiopia, while *H. dimidiata* instead remained in southern Africa and its origins can be traced back to 133 ± 8 kya. Finally, the most basal clade split from the hypothetical *Hirundo* ancestor (MRCA) 2494 ± 65 kya and encompasses *H. nigrorufa* and *H. atrocerulea*, also known as the “blue clade”. This clade originated 907 ± 45 kya with both species remaining in Central Africa. The *H. atrocerulea* ancestor can be traced back recently to 23 ± 0.5 kya (based on three modern mitogenomes). A putative date of origin for MRCA was estimated via molecular means and therefore has no reliance on previous fixed dates [6], as detailed in [17]. Moreover, the geographical location of the aforementioned individual was approximated based on the current distribution of closest species.

## 3. Discussion

### 3.1. Swallow Fingerprinting and Species Distribution

**Figure 2:**
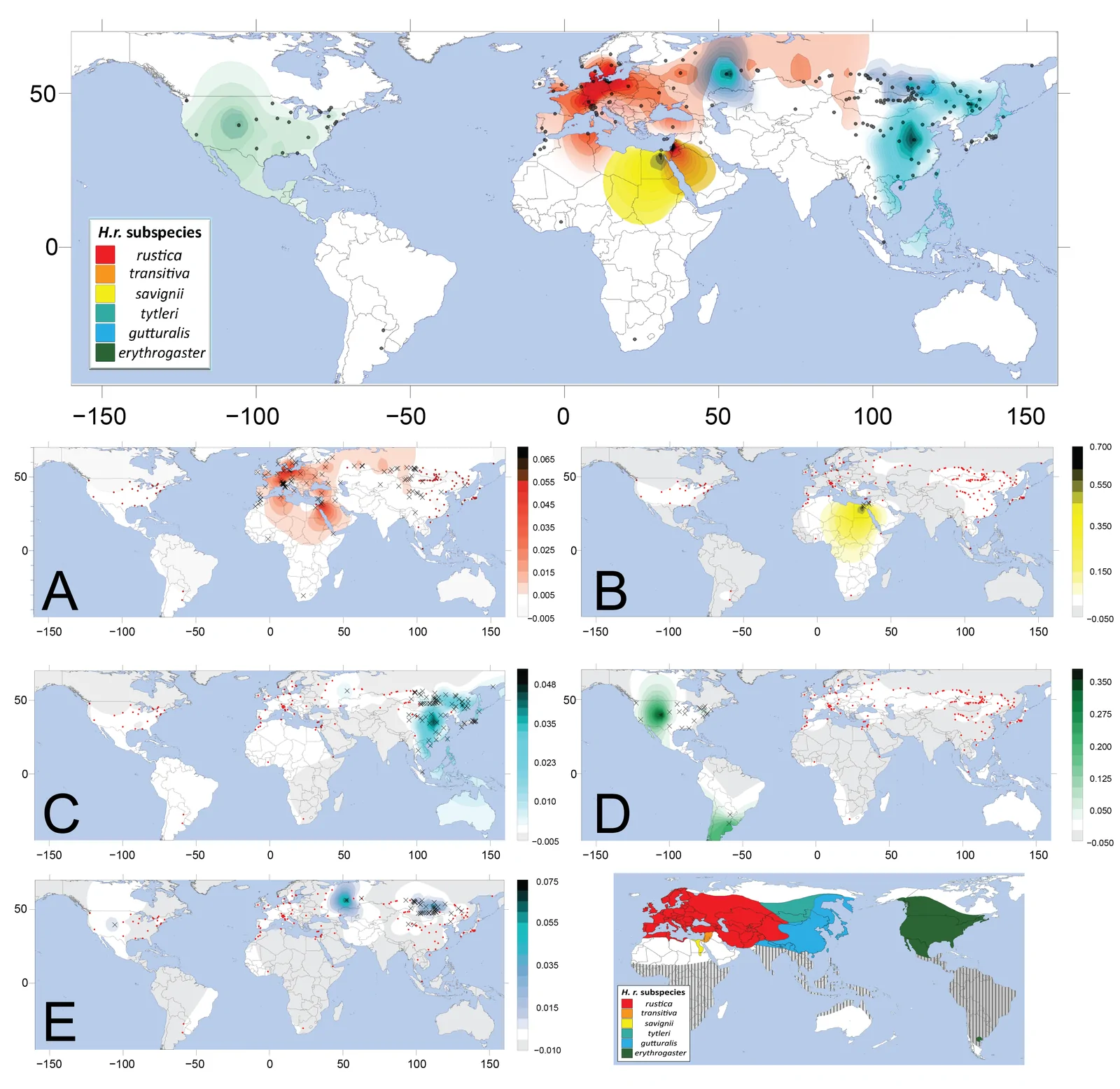
Haplogroup frequency distribution maps of main barn swallow haplogroups (**A**–**E**). The main map, above, is a composite of all subspecies distribution breeding ranges of all sampled individuals and database individuals of maps below, marked as maps (**A**–**E**) based on main haplogroup. Dots indicate the geographical locations of all sampled individuals. For each haplogroup, crosses indicate individuals of subspecies in question. Colour scale indicates frequency of haplogroup. Final map is the species distribution range as in [17].

The greatest hurdle in complete mitochondrial DNA mapping in swallows is the duplicated control region, also found in many avian species and especially in passerines [18]. In our case, the duplicated control region reads are indistinguishable from one another when not considering the 80 base pairs which are different in CR2 and the final 210 bps which are a long tandem repeat in CR2. This occurred for every individual and therefore CR2s were included just for phylogeny and not for dating branches as doubling the mutations in the CR regions would have incremented divergence times erroneously. Moreover, we uncovered a pattern in the CR mutations (Table S4) that are subspecies-specific and could be used to develop a cheap molecular method for subspecies determination that does not rely on phenotype or geography, useful for hybrid zones. GenBank and ENA were also mined for *H. rustica* mtDNA fragments, which can be used to produce a barn swallow SNP dataset to fingerprint samples at the subspecies level, or even better, at the sub-haplogroup level. These samples were from Project PRJNA323498, mainly 100 bp single-end reads previously digested with Msel and EcoRI which map on the mtDNA of most samples at rRNA 12s (829–907), *ND2* (4839–4923; 4924–4990), *COI* (5866–5931; 6215–6300), *COII* (7155–7302; 7310–7394), *ATP6* (8394–8459), *ND4* (11,166–11,251; 11,259–11,342), *cytb* (14,251–14,335), CR1 (15,140–15,224; 15,232–15,315) and CR2 (17,045–17,129; 17,137–17,220). This allowed us to correctly determine 89.3% of the samples (1280 individuals) at least to the subspecies level (Figure 2). This led to the discovery of two additional sub-haplogroup of *H.r. savignii*, named B4 and B5, where a rather large number (*N* = 27) of mitogenomes cluster in. This indicates that a potentially large part of the genetic variability in the subspecies breeding range is still underestimated. All other samples cluster into previously described major sub-haplogroups. Noteworthy individuals were two *H.r. erythrogaster* samples with *H.r. tytleri* mitogenomes present in Colorado, six *H.r. gutturalis* samples presenting either *H.r. rustica* or *H.r. tytleri* mitogenomes, six *H.r. rustica* samples presenting either *H.r. gutturalis* or *H.r. tytleri* mitogenomes and six *H.r. tytleri* individuals with *H.r. gutturalis* mitogenomes all sampled in the hybrid zones. Moreover, two *H.r. savignii* samples with *rustica* mitogenomes and four *H.r. transitiva* with *H.r. savignii* mitogenomes were found outside their distribution area, confirming the region as another hybrid zone. These samples were useful to uncover the maternal lineage of hybrid individuals. In fact, 486 individuals were hybrids and 463 (95%) could be identified at the subspecies level. Of the 683 single- or multi-gene accessions, 671 (98%) could be identified to the subspecies level, indicating that the resolution of our phylogeny is well structured and able to distinguish between subspecies using few SNPs.

### 3.2. Hirundo rustica, a Richer Phylogeny

**Figure 3:**
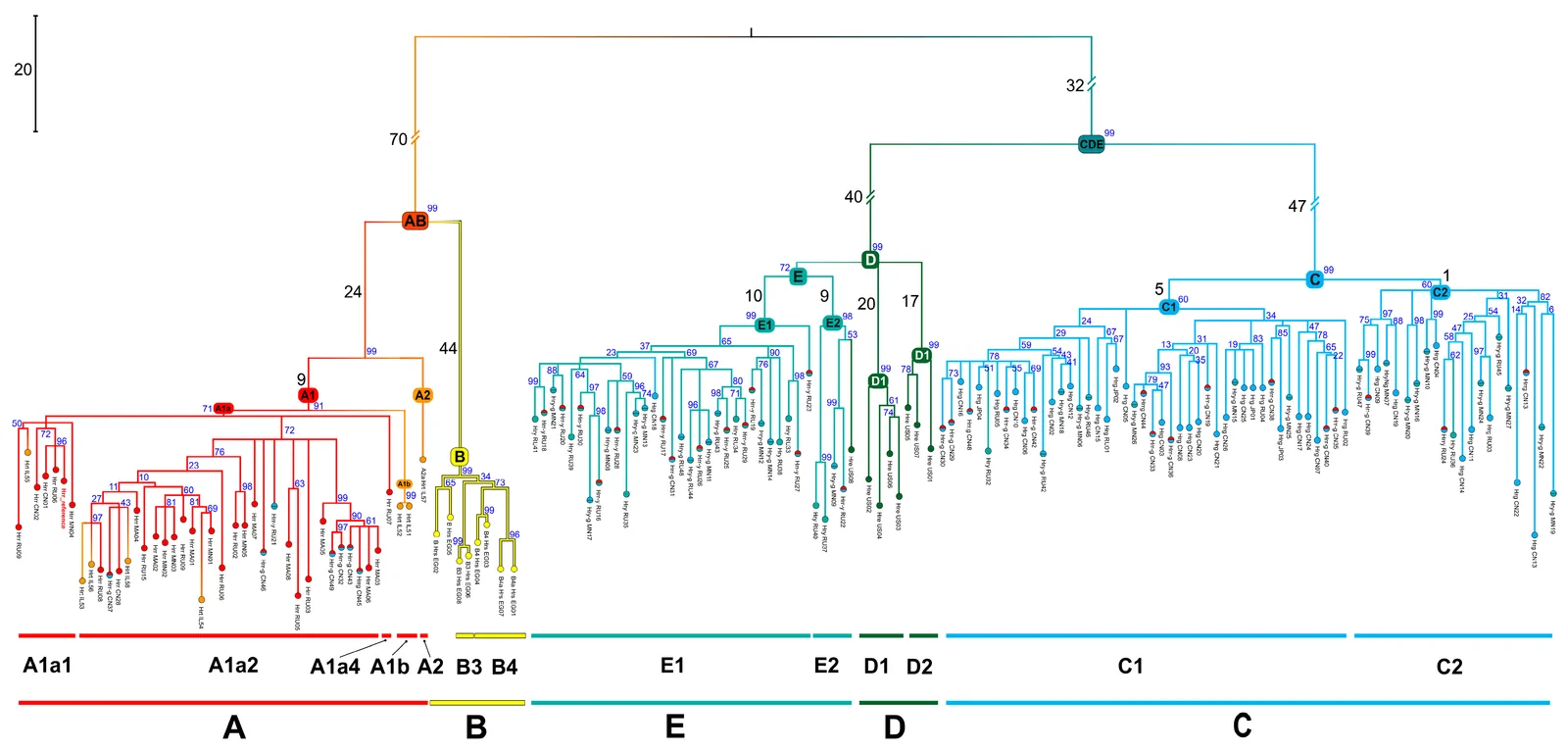
Maximum parsimony tree of *Hirundo rustica* mitogenomes. This tree encompasses only new complete samples added to the study. Colours indicate main subspecies. Numbers on main branches indicate the number of nucleotide substitutions while numbers at main nodes indicate bootstrap values.

Phylogenetic analysis of the 580 complete mitogenome coding regions (15,601 bp) revealed that the species is monophyletic deriving from a single ancestral female (HrAM). The addition of the new samples (Figure 3) confirms the same general phylogenetic structure depicted in previous findings [2,6,7], showing two major clades encompassing haplogroups A and B in one and C and D in the other. Due to a previous work [6] only harbouring four mitogenomes, a major overhaul in haplotype classification can be seen for haplogroup C (*N* = 72). For simplicity, the previously published four samples have been re-classified according to the new nomenclature described here, maintaining, however, the two main sub-haplogroup structure. One new feature of crucial importance in the phylogenetic tree which appears in this study is the new “main” haplogroup E; in fact, most *H.r. tytleri* were previously classified [2,6,7] as a sub-haplogroup of the American *H.r. erythrogaster* (haplogroup D) given the single SNP that defines the branch. That said, the current analysis has provided new insights, showing that the clade was distinct enough from the others, with π = 0.285% (±0.015%), so it has been named haplogroup E for simplicity’s sake (rather than D3 as previously theorised based on *cytb* sequences, [6]). Our results therefore provide an applied phylogenetic framework for understanding how demographic history has shaped the genetic landscape of *H. rustica*. Beyond the sole phylogenetic reconstruction, these findings illustrate how evolutionary processes—such as secondary contact, introgression, and lineage persistence—inform present-day genetic connectivity across migratory populations.

### 3.3. An Insight into H.r. transitiva

**Figure 4:**
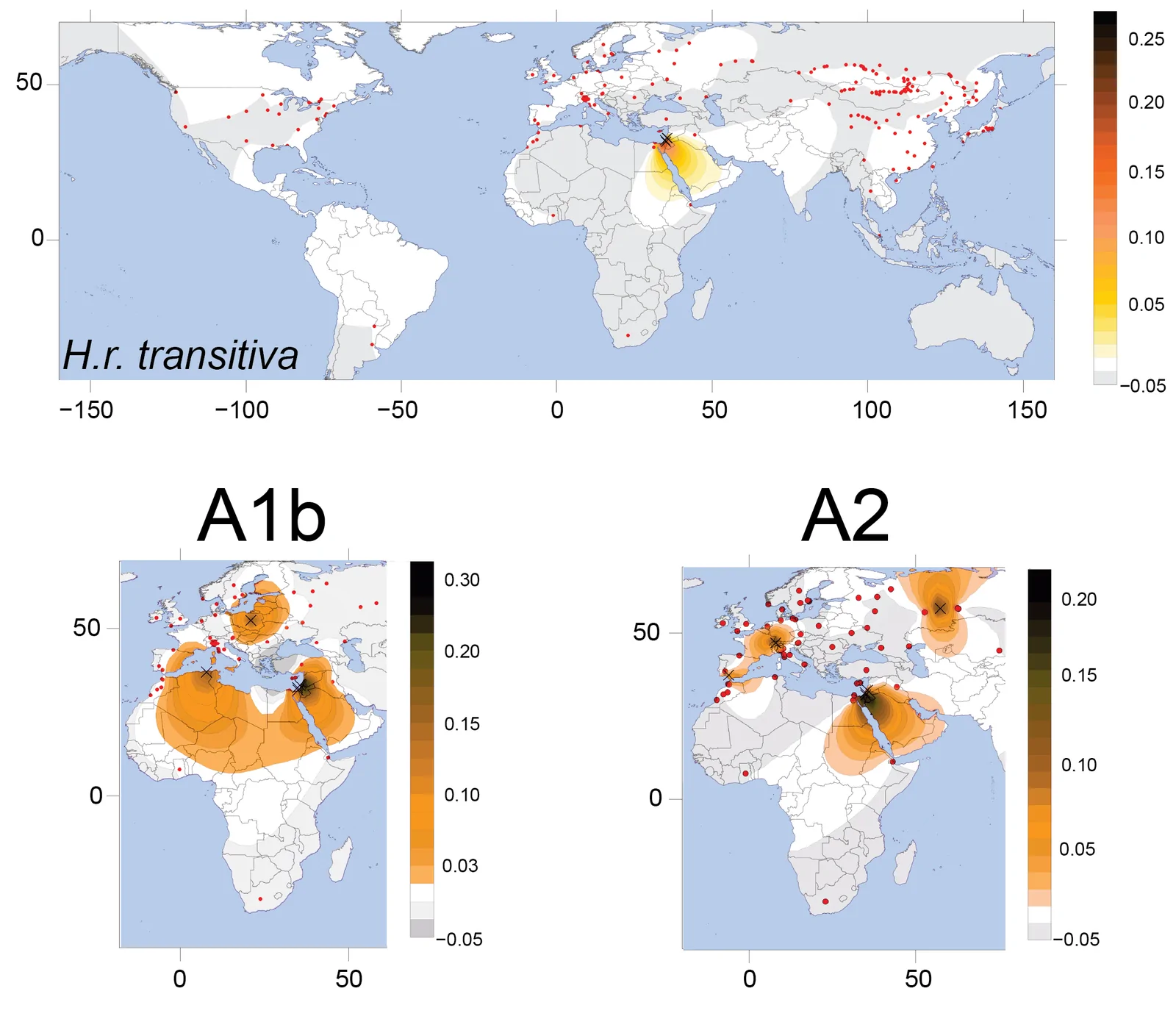
Haplogroup frequency distribution maps of *H.r. transitiva* individuals. The main map, above, indicates the distribution of all *H.r. transitiva* individuals, while the maps below are specific to haplogroups A1b and A2. Dots indicate the geographical locations of all sampled individuals. Crosses indicate individuals of subspecies in question. The colour scale indicates the frequency of the haplogroup in the given map.

Having been previously defined by only four entire *H.r. transitiva* (based on morphology and behaviour) mitogenomes from Israel, haplogroup B could not be clearly and with 100% certainty be defined as the *H.r. transitiva* mitogenome. This was further complicated by the knowledge of admixture in the area and further partially refuted by using *cytb* sequences [6,7]. This analysis showed that Egyptian *H.r. savignii* birds harboured the same DNA as the four *H.r. transitiva* specimens already present in this haplogroup. The addition of eight complete *H.r. savignii* sequences (sampled in Egypt with correct phenotypic traits) [14] confirms the four noted haplogroup B individuals from Israel are therefore of *H.r. savignii* maternal origins and are present in the previously postulated hybrid zone in the Sinai Peninsula, a genetic and geographic crossroad between the three subspecies in the area. The behaviour and phenotype of *H.r. transitiva* further reflects this, being non-migratory with a rufous breast with reddish underparts, which make it more similar to *H.r. savignii* than *H.r. rustica*; this indicates an intermediate phenotype between *H.r. rustica* and *H.r. savignii*. We therefore confirm haplogroup B as *H.r. savignii* like *cytb* analysis suggested. All *H.r. transitiva* individuals added in this study are classified in haplogroup A. Haplotypes A1b and A2 (Figure 4) present a peculiar finding. These are mainly *H.r. transitiva* individuals with the exception of a Polish and Algerian *rustica* (A1b) and a Spanish and Italian *rustica* (A2). Both these haplogroups have a rather noteworthy number of defining private mutations (11 and 17, respectively), which indicate their older ancestral origin than that of the other *H.r. rustica* haplogroups. This can be a hint towards a subspeciation event (or two) (nucleotide diversity values, π ≈ 0.2–0.3%). Obtained nucleotide diversity values are in the range of those seen for other bird species and subspecies [6,19,20], with A1b being on the lower end π = 0.175% and, on the other hand, A2 being in line with the values of another subspecies (π = 0.294%). Furthermore, five samples from Israel identified via phenotypical characteristics [5,14] and *cytb* and CR fragments cluster within haplogroups A1b and A2, lending further credence to this hypothesis. In addition, A2 diverged pre-LGM and is likely the ancestral *H.r. transitiva* mitogenome. This lineage either stayed in the Levant region or migrated south during the LGM and returned to the study area after the Younger Dryas until the present day, as seen in other migratory birds [21,22,23,24]. We would therefore expect to see this pattern in the population structure analysis of nuclear genomes, which would help discriminate between recurrent gene flow or incomplete lineage sorting. Gene flow still occurs between migrating *H.r. rustica* and *H.r. transitiva* individuals, and therefore neither has been isolated enough to properly diversify, or as can be hypothesised by the four sub-haplogroups of B in *H.r. transitiva* individuals, haplogroup A has replaced the previously present haplogroup B (still present in Egypt).

### 3.4. Resettling of the Old World

The east-to-west trans-Beringian colonisation direction of *H.r. tytleri* has been postulated to have occurred with the arrival of humans and their constructions in the Siberian area [25]. The close relationship between the predominantly Asian haplogroup E and the American haplogroup D is compatible with different phylogeographic scenarios. These include the previously proposed back-migration from North America to the Palearctic [1,2,6,7,9,10,17], possibly occurring in two main dispersion events, those of haplogroup E1 and E2, as well as divergence from an ancestral Asian population followed by colonization of North America. The present mitochondrial data alone may not unambiguously resolve the direction of dispersal.

Haplogroups E1 and E2 share a single SNP in common in the coding region at the E root, calculated to have diverged around 58 kya and have greatly diverged since without geo-specificity as both are found throughout the distribution range.

The presence of an E mitogenome in an American individual is consistent with historical or more recent gene flow between Asian and American populations. Given the considerable number of ND2 and COI mutations on the DE node (7 and 5, respectively), even partial sequences present in the database could be clearly identified (Figure S1) at the sub-haplogroup level (albeit they could not be used for divergence time estimations). Furthermore, observed overall, patterns of mitochondrial diversity are consistent with historical population expansions followed by localised secondary contact among Eurasian lineages, reflecting repeated episodes of connectivity and isolation during Late Pleistocene climatic oscillations.

### 3.5. Hirundo rustica Hybrids

**Figure 5:**
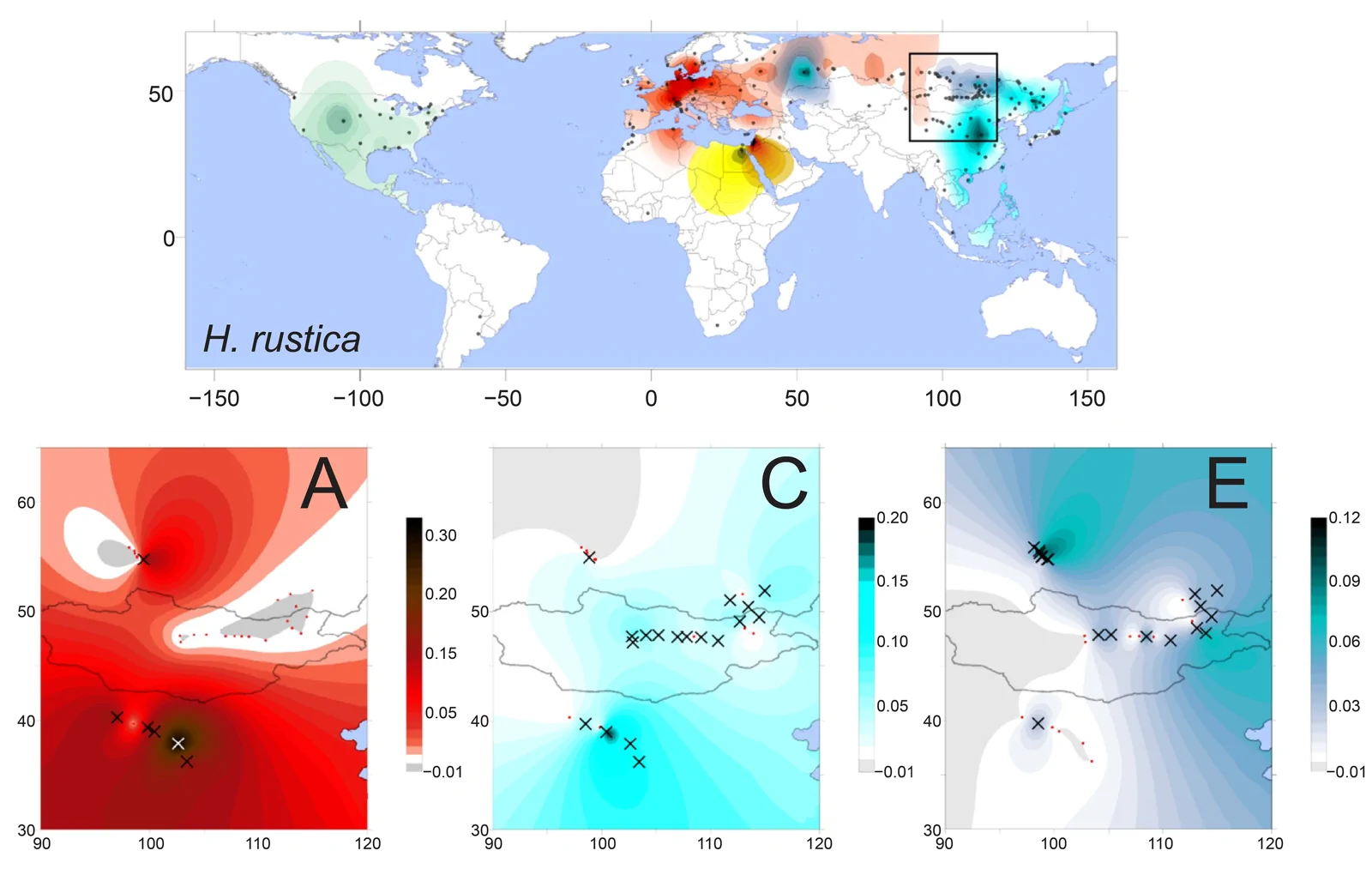
Haplogroup frequency distribution maps of *Hirundo rustica* hybrids. Letters ACE represent, respectively, the distribution of *H.r. rustica*, *H.r.gutturalis* and *H.r. tytleri* distributions in the same hybrid zone transect of the map. The square in the large map above is the studied area in the three maps below. Dots indicate the geographical locations of all sampled individuals. Crosses indicate individuals of subspecies in question. The colour scale indicates the frequency of the haplogroup in the given map.

Unlike the migratory divide between *rustica* and *gutturalis* in Western China and that of *tytleri* and *gutturalis* in Northern Mongolia and at the border between Russia and China, the hybrid samples from the Irkutsk Oblast region of Russia (*rustica-tytleri* hybrids) show a significant skew from the expected haplogroup distribution (Figure 5). These have a majority of *H.r. tytleri* mitogenomes indicating that maternal lineages in the hybrid regions are mostly *H.r. tytleri*. This could suggest a sexual selection preference of female *H.r. tytleri* for male *H.r. rustica* individuals, as can be seen by the hybrid phenotypes, which are intermediate at the contact zones. This, however, goes against what has been previously studied, at least, just between *rustica* and *tytleri* and only from the mitogenome point of view, where patterns of sexual selection as reproductive barriers were uncovered [26,27].

### 3.6. Before Barns: Uncovering the Swallow’s Past

**Figure 6:**
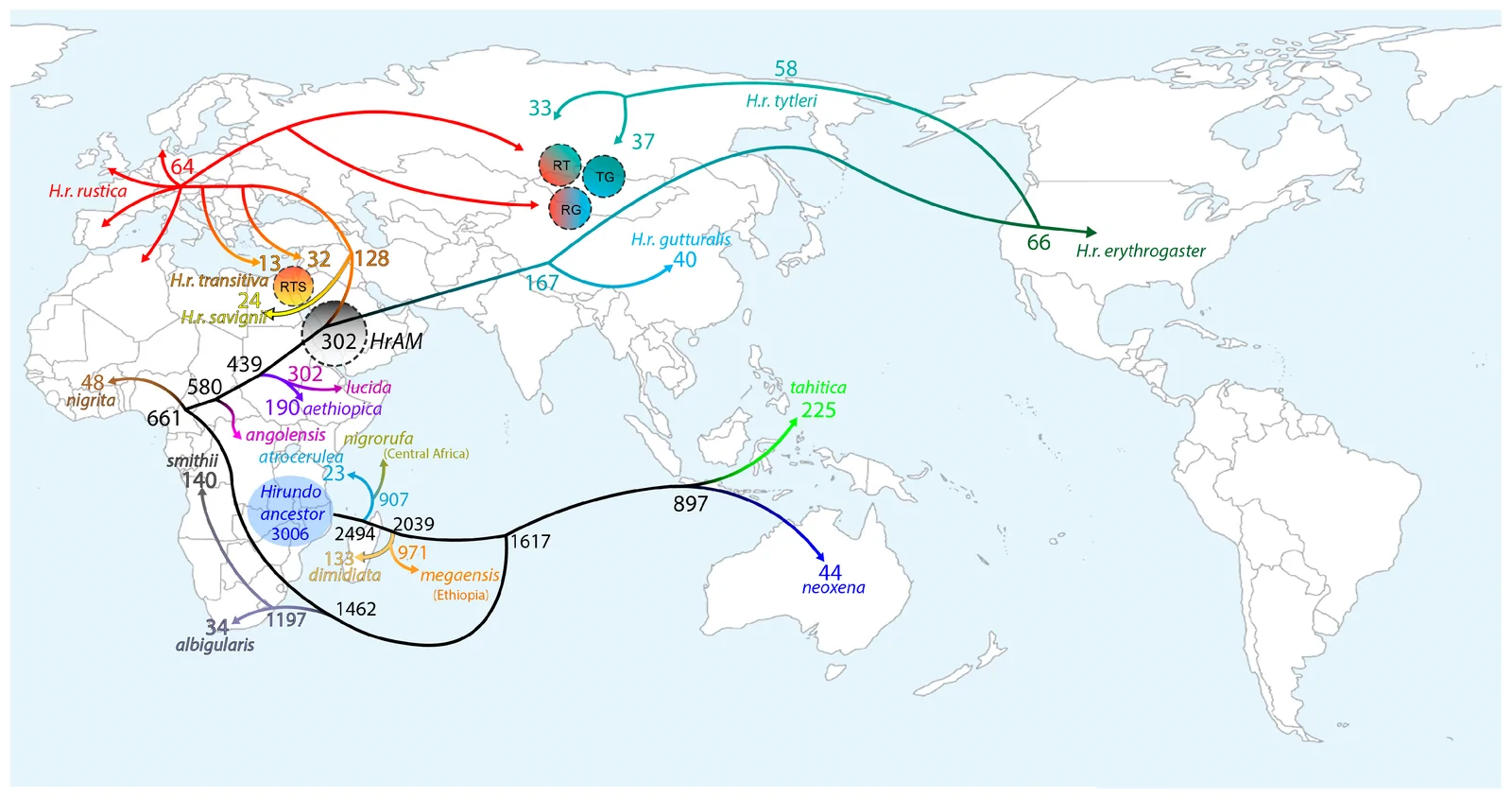
A model for the geographical and temporal spread of swallows. Map showing divergence times, in thousands of years ago (kya), of hypothetical splits and diffusion routes of *Hirundo* species and barn swallow haplogroups. The dashed grey circle indicates the possible homeland of HrAM, and the blue circle indicates the putative area of origin of all *Hirundo* species’ most recent common ancestor (MRCA). All other dashed circles indicate zones where two subspecies are currently found (RTS, *rustica-transitiva-savignii*, RT, *rustica-tytleri*; TG, *tytleri-gutturalis*; and RG, *rustica-gutturalis*), indicating recent admixture between subspecies. Note that haplogroup A arrows are not related to any specific haplogroup but simply show the vast expansion of the subspecies.

We obtained new and updated molecular based ages for all nodes in the barn swallow phylogeny using Bayesian age estimates (Table S4). With the expansion of the previous major haplogroups and the addition of the new haplogroup E, we have found older nodes present in the East, thus suggesting that the ancestral barn swallow mitogenome progenitor (HrAM) should be at 302 ± 8 thousands of years ago (Figure 6), close to the higher upper boundary of the confidence interval of the previous study [6]. This event could have taken place in northeastern Africa (Sudan/Ethiopia/Somalia) as the closest species (*H. ethiopica* and *H. lucida*) are still present there today (Figure 6) [28,29]. Moreover, given our extensive phylogenetic analysis, we trace back the origin of all *Hirundo* species to a common mitochondrial ancestor ~3 Mya in what was probably southern Africa, as supported by the fact that the most basal species of the genus are still present there. From here, the phylogenetic divergence follows a northward patten of colonisation, with more recent species currently located more to the north than species which split before, up to the worldwide expansion of *H. rustica*. We note that the Pacific clade branched off 1.6 Mya (in line with what can be seen from nuclear data, [30]) and reached southeast Asia and Australia long before *H. rustica* expanded. This most likely occurred via island hopping [30,31]. The three new complete mitogenomes, *H. nigrorufa*, *H. megaensis* and *H. lucida*, fall into previously described clades with other species as in previous *ND2* and *cytb* studies [2,7]. One major change to the phylogeny is the placement of *H. albigularis* which was previously considered basal to the pacific clade and the *Hirundo* clade [7], probably because of incomplete data, while it is now considered a sister species of *H. smithii.* The latter species is also interesting as two main subspecies have been described, *H.s. smithii*, present throughout Africa, and *H.s. filifera*, present in Asia [32]. Our analysis shows two major haplogroups which separated from each other around 140 kya, i.e., at a time comparable to that of the divergence of *H. rustica* subspecies. These two haplogroups are also indicative of the presence of ancestral African mitochondrial DNA now typical of the Asian subspecies.

## 4. Materials and Methods

### 4.1. Samples Analysed

We obtained a total of 168 barn swallow mitogenomes (Table S5) by extracting mitochondrial reads from genomic Bioprojects (PRJNA304409, PRJNA323498, PRJNA545868, PRJNA909772, PRJNA1117501) [13,14,27,33,34] using a modified automated pipeline [35]. These new mtDNAs include individuals from six subspecies: 30 *H.r. rustica* from Europe, Asia and North Africa; 8 *H.r. savignii* from Egypt; 8 *H.r. transitiva* from Israel; 35 *H.r. gutturalis* from Russia, China and Japan; 10 *H.r. erythrogaster* from the U.S.A.; and 11 *H.r. tytleri* from Russia. Moreover, mitogenomes from three hybrid populations were obtained: 21 *rustica-gutturalis* hybrids from China; 16 *rustica-tytleri* hybrids from Russia; and 29 *tytleri-gutturalis* hybrids from Mongolia and Russia (Table S5). In addition to barn swallow subspecies, we obtained the mitogenomes of 12 other *Hirundo* species (*H. atrocaerulea*, *H. nigrorufa*, *H. megaensis*, *H. dimidiata*, *H. tahitica*, *H. neoxena*, *H. albigularis*, *H. smithii*, *H. nigrita*, *H. angolensis, H. lucida*, *H. aethiopica*). Remaining sequences (411 entire mitogenomes) were downloaded from Genbank, Bioproject (PRJEB51610) [6]. Other deposited complete mitogenomes were found to be erroneous in a previous work [6] and were not included, aside from mitogenome KX398931 (*H.r. erythrogaster*, [36]), whose small NUMT was considered as a missing segment. Furthermore, only for fingerprinting and distribution analysis, we downloaded 1440 fastq files belonging to Bioproject PRJNA323498 [14] and 1509 accessions of barn swallow genes belonging to 683 different individuals. These were then aligned to the *H.r. rustica* reference sequence (MZ905359) [17] using the fast Geneious mapper (v.2024.0.5). Variant calling was performed using the Geneious algorithm and samples were classified by hand by comparing individuals to the complete mtDNA alignment. Note that all partial sequences were only used for internal classification and not for any phylogenetic or temporal analysis.

### 4.2. Mitochondrial Read Extraction and Mapping

Mitogenome reads were extracted from genomic reads using an in-house pipeline. Data were downloaded from the sequence read archive (SRA) by filtering for the term *Hirundo* as paired end fastq files, NGS long reads or complete genomic scaffolds. The raw single file samples were firstly loaded into Geneious Prime v.2024.0.5 (https://www.geneious.com, Dotmatics, Boston, MA, USA) and filtered by removing duplicate sequences using Dedupe from BBTools, then aligned and mapped to the barn swallow reference sequence (MZ905359) [17] using the Geneious mapper. On the other hand, samples with paired end sequences were run through an in-house pipeline for swallow analysis. Adapters and low-quality ends with phred score below 30 (99.9% likely) were removed with TrimGalore v.0.6.10 [37], and proper adapter flags were set for each dataset. Trimming efficiency was then checked using FastQC v.12.0 [38] to determine quality of remaining reads. Files were then aligned and mapped to the barn swallow reference sequence (MZ905359) using bwa-mem v. 0.7.17 [39]. Resulting BAM files were uploaded to Geneious Prime for manual curation. To avoid nuclear mitochondrial insertions (NUMTs), all reads showing excessive divergence (>6 mutations from reference within 100 bp windows) were excluded, and regions with ambiguous coverage were manually checked. Consensus sequences were verified by visual inspection of read alignments to ensure authenticity of mitochondrial origin. Variant calling was performed using the Geneious algorithm when at least 70% of reads shared a mutation; otherwise, they were considered heteroplasmies. Control region mutations usually mapped twice, once in CR1 and once in the repeated portion of CR2. In addition, the number of repeats at the end of CR2 were uncountable due to the limitations of short reads mapping and therefore were aligned to the reference sequence without repetition indels. Consensus for each file was then output as a fasta file and aligned with Genbank entries using mega x v.11.0.11 [40].

### 4.3. Phylogenetic Analyses and Age Estimates of MtDNA Haplogroups

Nucleotide diversity (π) of the complete coding region (~15.6 kb) was estimated for each haplogroup and subspecies using DnaSP v6.12.03. Nucleotide diversity was calculated as the average number of nucleotide differences per site between all possible pairs of sequences within each group, using aligned mitochondrial genome sequences after excluding sites containing gaps or missing data. To further evaluate the effect of unequal sample sizes, a rarefaction analysis was performed by randomly selecting 40 haplogroup A individuals, including six *H.r. transitiva* specimens to preserve the observed subspecies representation (Table S2). Pairwise genetic distances were recalculated using the rarefied dataset and compared with those obtained from the complete dataset (Table 1).

The complete maximum parsimony (MP) tree (Figure S1) was built by modifying a previously published mtPhyl v. 5.003 [41] script to account for new samples [6], haplogroups and control region mutations. New haplogroups and sub-haplogroups were defined when two samples shared a new, non-recurring mutation in the coding region of the mtDNA. Indels were not considered for tree construction. The Western house martin, *Delichon urbicum* (NC_050298), was used as an outgroup. Tree topology was confirmed also via a 1000 bootstrap MP tree in mega x v.11.0.11. Ages of haplogroups and sub-haplogroups were determined via molecular methods by performing Bayesian estimations using beast v.2.7.7 [42] under the HKY (κ = 2, estimated frequencies) nucleotide substitution model (gamma-distributed rate heterogeneity with 8 categories, 0.5 shape and estimated 0.5 proportion of invariant sites) [43] with a relaxed clock log-normal value of 0.0245 s/s/Myr (or one mutation every 2616 years) as in [6,17] to avoid node restraints. Current population size was set to a mean estimate [44] value of 388 million individuals (uniform distribution, 487 M upper, 290 M lower). MCMC chain length was set to 50,000,000 iterations, with samples drawn every 1000 steps after a burn-in of 10% using both log combiner and tree annotator (as in [45]). Bayesian skyline plots were constructed using median tree heights and 2000 bins using Tracer v.1.7.2 [46]. Haplogroup expansion maps were built using surfer v 19.1.189 (Golden Software, Inc., Golden, CO, USA, www.goldensoftware.com/) using the point kriging method (Figure 2) and overlayed on a world map.

## 5. Conclusions

This study presents the most extensive mitogenomic analysis of the barn swallow to date, encompassing 580 complete high-quality mitochondrial genomes representing all six recognised subspecies. The phylogeny of the genus *Hirundo* supports the postulated northeastern African origin of *H. rustica* and resolves its position within its Afro-Palearctic relative family tree. We identify a clear phylogeographic structure comprising five well-supported haplogroups. Among these, the newly identified haplogroup E, largely restricted to *H.r. tytleri*, adds complexity to the mitochondrial diversity of *H. rustica* and suggests the presence of previously unrecognised evolutionary lineages. Our time-calibrated phylogenies suggest that divergence among the major haplogroups may be associated with Late Pleistocene climatic oscillations.

Patterns of haplotype diversity and demographic reconstructions support historical population expansions and secondary contacts, particularly in the Palearctic, and are consistent with previous evidence from nuclear genomic data, suggesting recurrent gene flow and incomplete lineage sorting.

Overall, our findings resolve key aspects of the evolutionary history of *H. rustica* and provide a foundation for future integrative research. Combining mitogenomic and nuclear genomic data will be essential to disentangle the relative contributions of historical demography, selection, and stochastic processes to present-day genetic diversity. This study provides a robust mitogenomic framework for a model migratory passerine and facilitates the integration of evolutionary, demographic, and ecological perspectives relevant to conservation management. More broadly, linking genomic patterns with ecological processes can help elucidate connectivity, adaptation, and population persistence under environmental change.

## Figures and Tables

**Table 1:** Nucleotide diversity estimates (%) within and between barn swallows belonging to different haplogroups and from different geographic areas with their respective SD. Shading goes from green, lowest diversity to red, highest diversity. Intragroup nucleotide diversities (π) are on the diagonal. HrAM refers to the *Hirundo rustica* ancestral mitogenome node (all samples).

	*rustica*	*transitiva*	*savignii*	*gutturalis*	*erythrogaster*	*tytleri*	*HrAM*
*rustica n* = 389	0.13%	0.16%	0.53%	1.20%	1.20%	1.24%	0.39%
	±0.002%	±0.014%	±0.015%	±0.011%	±0.019%	±0.015%	±0.019%
*transitiva n* = 44		0.19%	0.50%	1.20%	1.20%	1.24%	0.41%
		±0.024%	±0.039%	±0.029%	±0.049%	±0.040%	±0.021%
*savignii n* = 12			0.08%	1.19%	1.19%	0.50%	0.67%
			±0.008%	±0.068%	±0.117%	±0.039%	±0.020%
*gutturalis n* = 72				0.16%	0.71%	0.73%	1.02%
				±0.006%	±0.024%	±0.020%	±0.017%
*erythrogaster n* = 24					0.20%	0.29%	1.02%
					±0.024%	±0.015%	±0.019%
*tytleri n* = 39						0.17%	1.05%
						±0.021%	±0.018%
*HrAM n* = 580							0.54%
							±0.023%

## Data Availability

All mitogenome sequences generated in this study have been deposited in GenBank under accession numbers PX422303-PX422462.

## Supplementary Materials

The following supporting information can be downloaded at: https://www.mdpi.com/article/10.3390/ijms27167433/s1.
