# Supplementary material for: Mitogenomes Reveal Novel Phylogeographic Patterns and Temporal Evolutionary History of Barn Swallows and Close Relatives
Source: Int J Mol Sci. 2026 Aug 20;27(16):7433. doi: 10.3390/ijms27167433 (PMC13513802; doi:10.3390/ijms27167433)
Supplement: Supplementary file 1 [file ijms-27-07433-s001.zip › Lombardo Supplementary Material_review.pdf]

Communication

Mitogenomes Reveal Novel Phylogeographic Patterns and Temporal Evolutionary History of Barn Swallows and Close Relatives

Gianluca Lombardo [1,2,*], Guido Roberto Gallo [1], Andrea De Benedictis [2], Marta Cavallini [2], Giulio Formenti [3] and Giorgio Binelli [2]

[1] Department of Biosciences, University of Milan, 20133 Milano, Italy; guido.gallo@unimi.it
[2] Department of Biotechnology and Life Sciences (BDSV), University of Insubria, 21100 Varese, Italy; giorgio.binelli@uninsubria.it (G.B.)
[3] The Vertebrate Genome Laboratory, The Rockefeller University, York Ave 1230, New York, NY 10021, USA
* Correspondence: gianluca.lombardo@unimi.it

Supplementary Material

Supplementary Figures

Figure S1. Detailed maximum parsimony phylogeny of *Hirundo rustica* mitogenomes. This tree was built using the entire mitogenome coding-region and was rooted using *D. urbicum*. Main haplogroup and sub-haplogroup affiliations are shown. Sub-haplogroups were named only when encompassing at least two haplotypes. Subspecies affiliations are according to the colours in the legend. Mutations, relative to HrrRS, are transitions unless a base is explicitly indicated. Suffixes indicate transversions (to A, G, C, or T). Reversions are marked with "@" and recurring mutations are underlined. Both sample ID names (in green) and IDs employed in phylogenetic analyses are provided. Heteroplasmic positions are shown below sample names.

EXCEL FILE

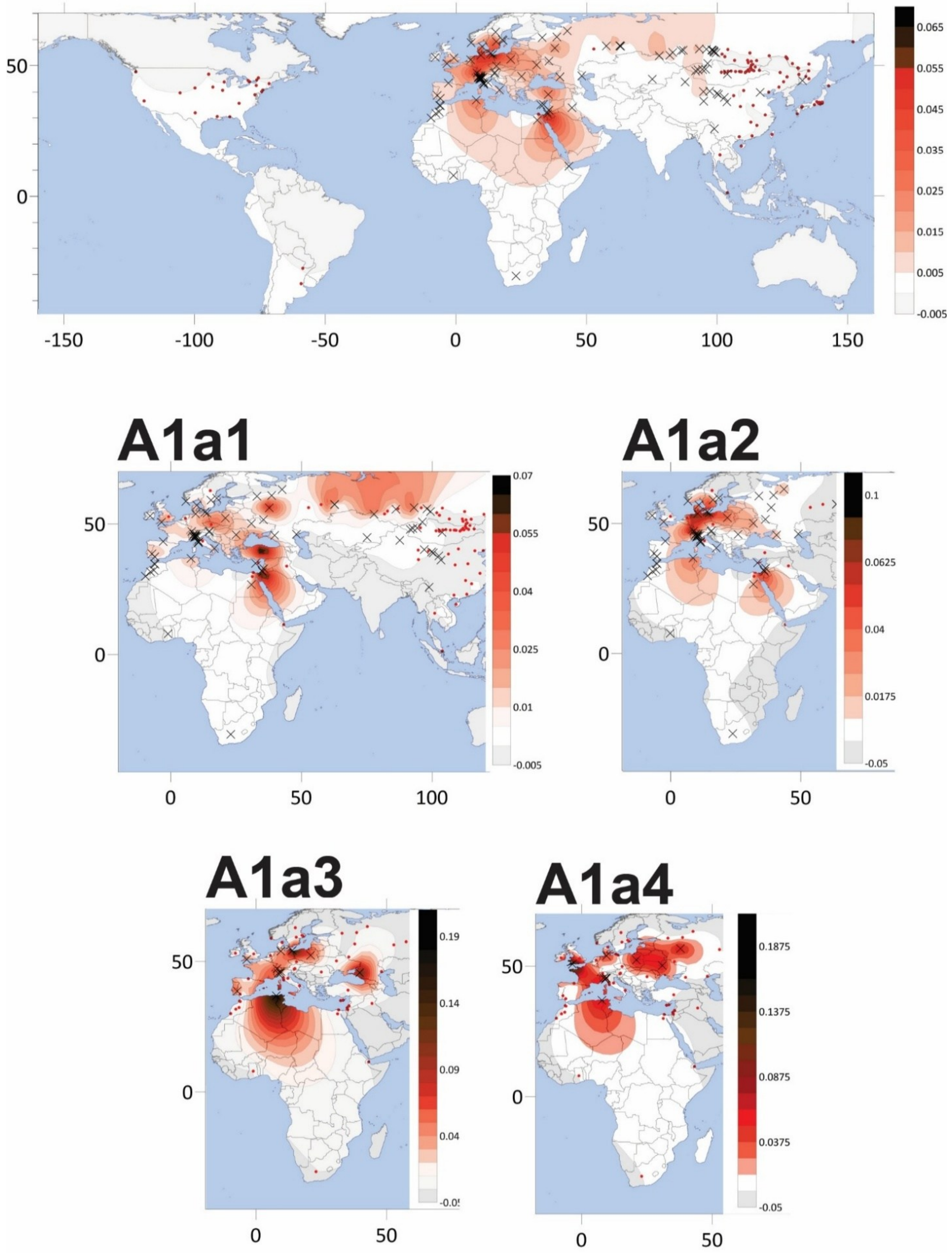
A1a1
A1a2
A1a3
A1a4

Figure S2. Haplogroup frequency distribution maps of *Hirundo rustica rustica* main haplogroups. Dots indicate the geographical locations of all sampled individuals; Crosses indicate individuals of subspecies in question. Colour scale indicates frequency of haplogroup in the given map.

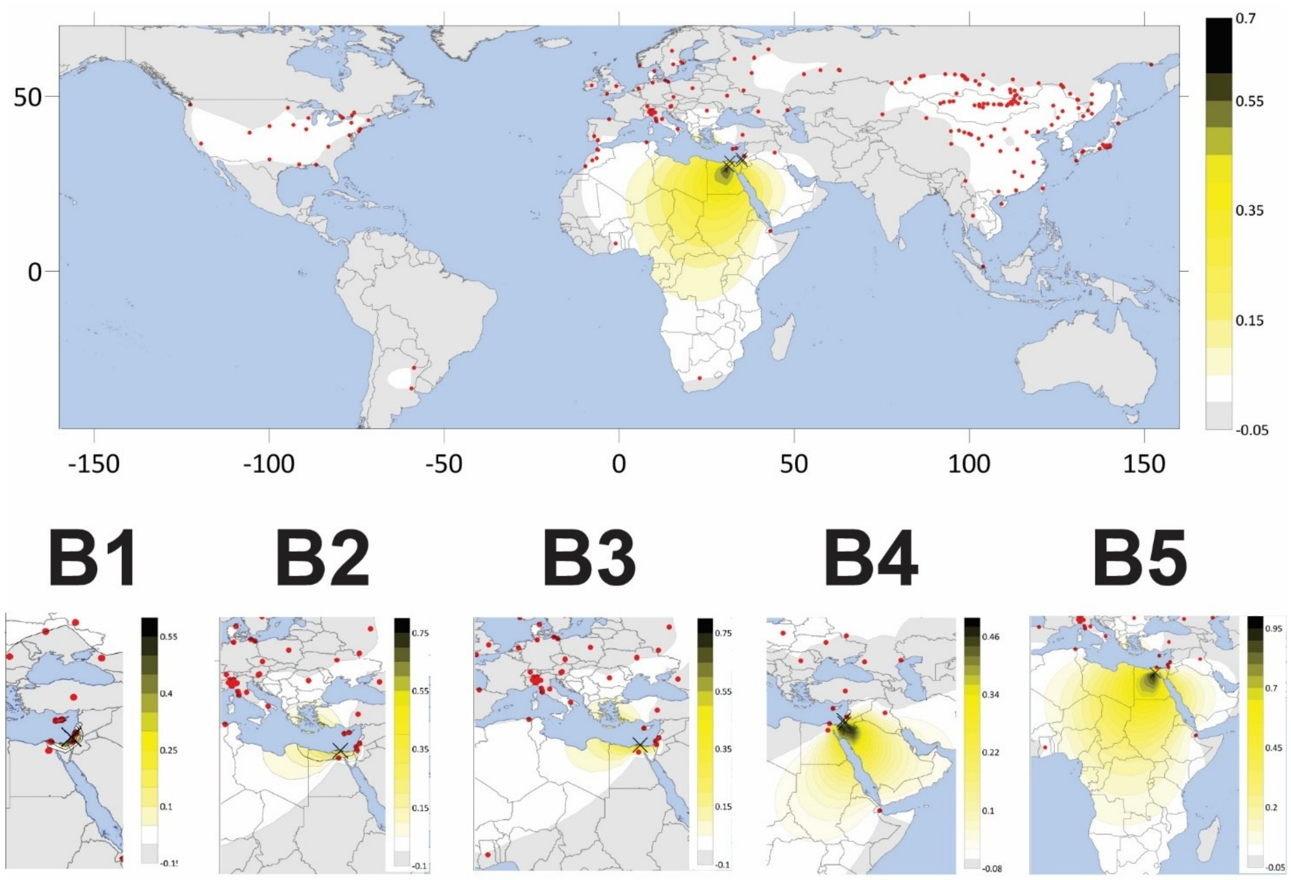

Figure S3. Haplogroup frequency distribution maps of *Hirundo rustica savignii* main haplogroups. Dots indicate the geographical locations of all sampled individuals; Crosses indicate individuals of subspecies in question. Colour scale indicates frequency of haplogroup in the given map.

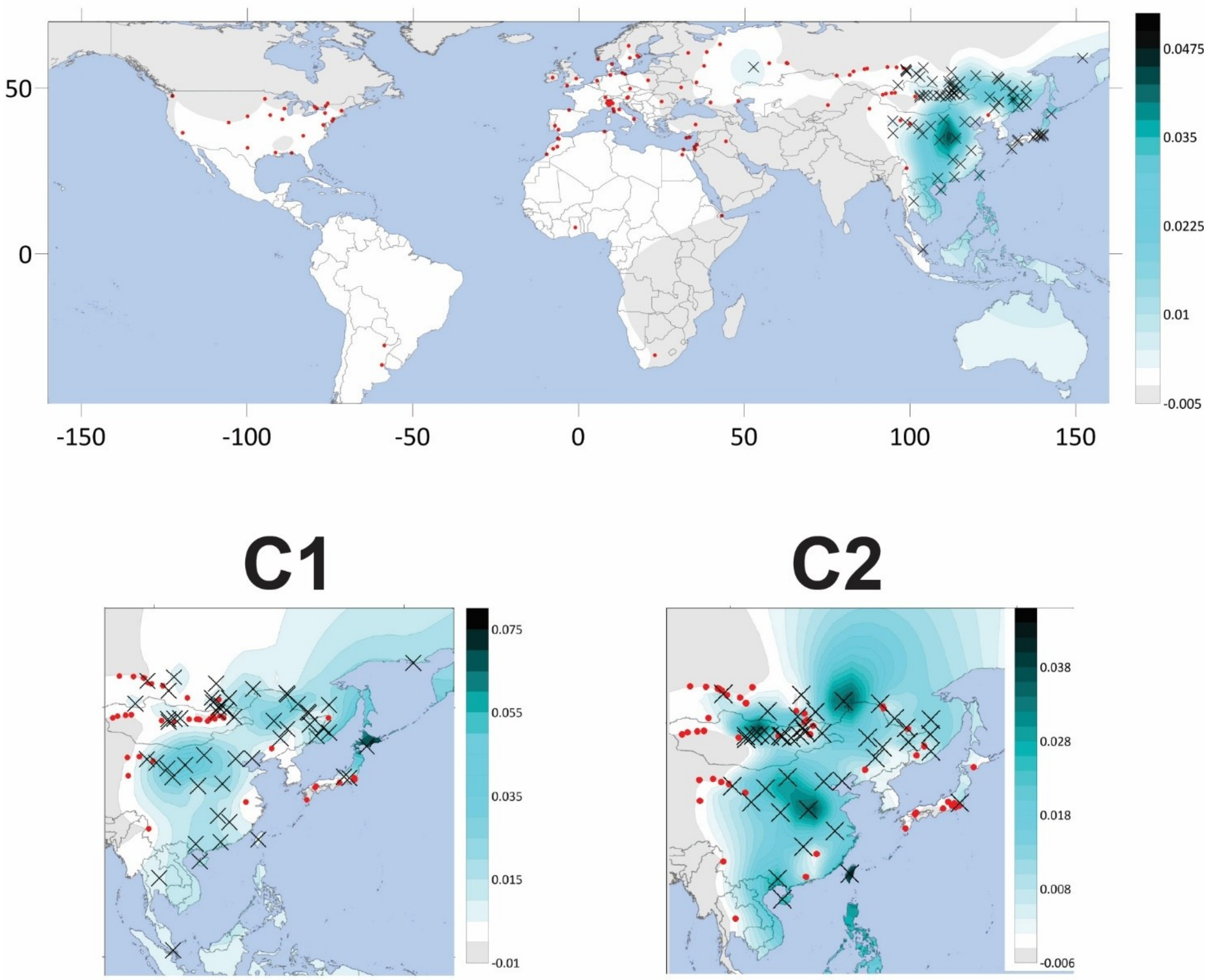

Figure S4. Haplogroup frequency distribution maps of *Hirundo rustica gutturalis* main haplogroups. Dots indicate the geographical locations of all sampled individuals; Crosses indicate individuals of subspecies in question. Colour scale indicates frequency of haplogroup in the given map.

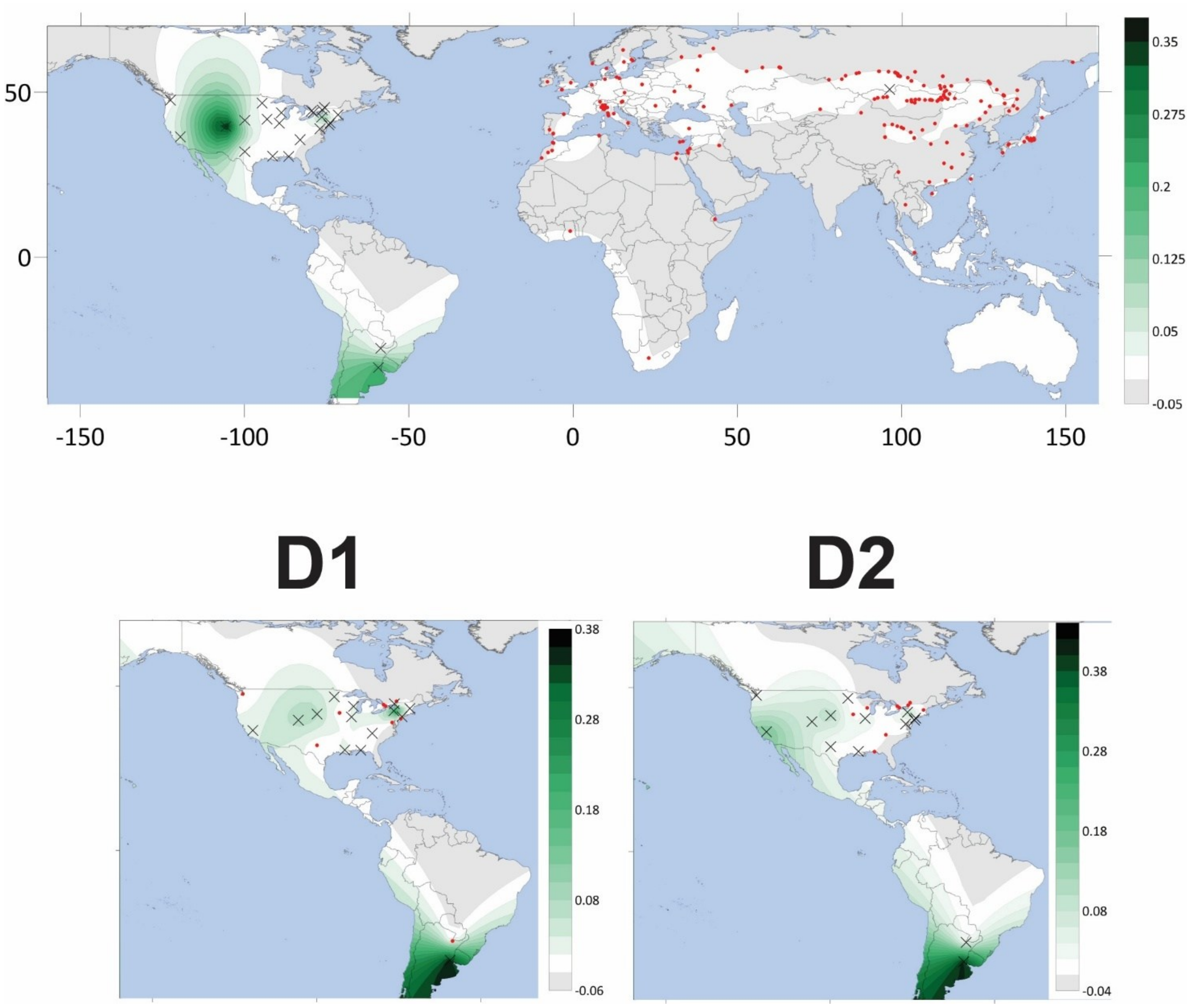

Figure S5. Haplogroup frequency distribution maps of *Hirundo rustica erythrogaster* main haplogroups. Dots indicate the geographical locations of all sampled individuals; Crosses indicate individuals of subspecies in question. Colour scale indicates frequency of haplogroup in the given map.

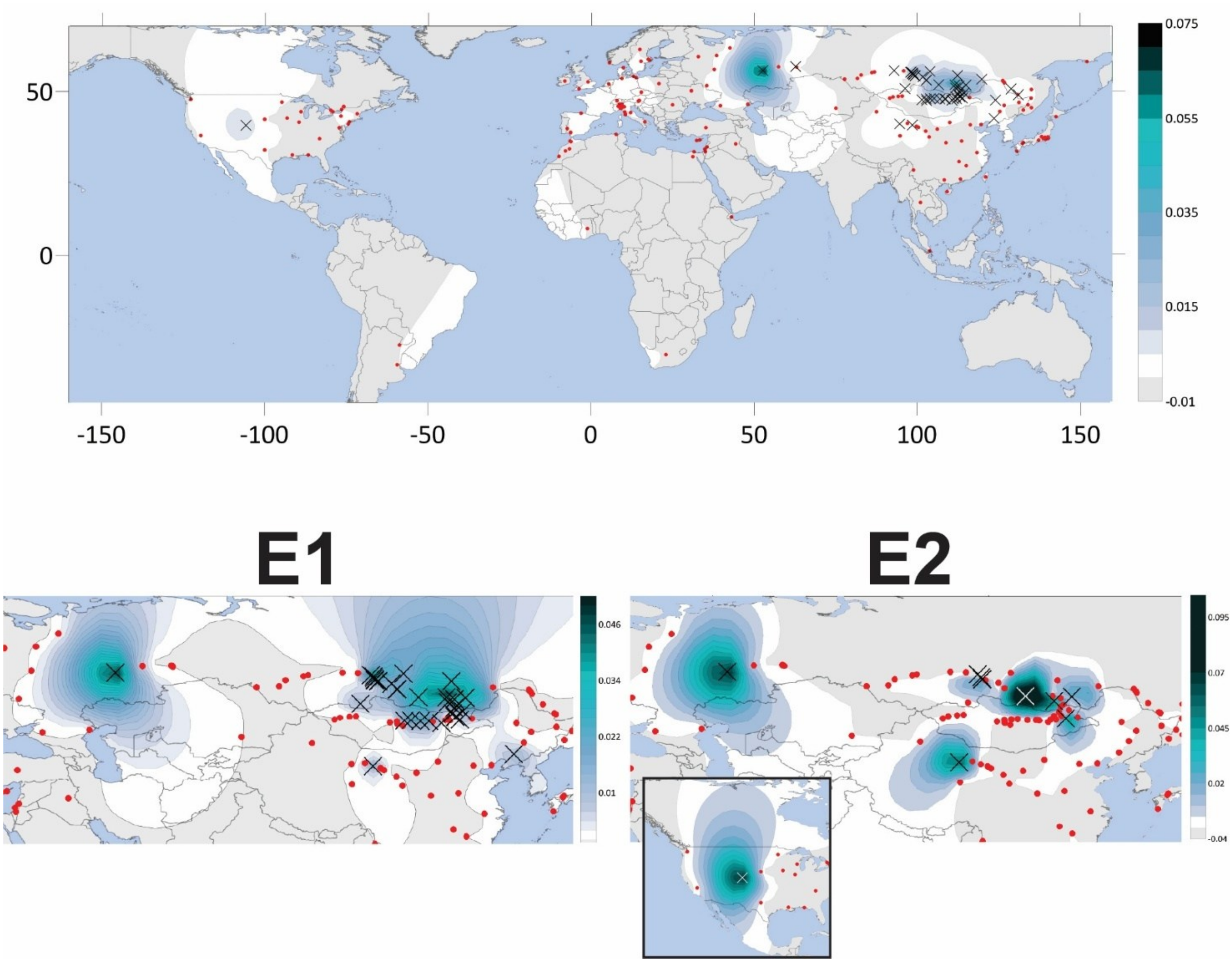

Figure S6. Haplogroup frequency distribution maps of *Hirundo rustica tytleri* main haplogroups. Dots indicate the geographical locations of all sampled individuals; Crosses indicate individuals of subspecies in question. Colour scale indicates frequency of haplogroup in the given map.

Supplementary Tables

Table S1. Extended nucleotide diversity (%). Within and between barn swallow main haplogroups with their respective SD. Shading goes from green, lowest diversity to red, highest. Intragroup nucleotide diversities (π) are on the diagonal.

	A	B	C	D	E	HRAM	OUT	A HRR	A HRT	A1B	A2	AB	CDE
A	0.153 ±0.002	0.530 ±0.012	1.203 ±0.010	1.198 ±0.018	1.242 ±0.014	0.390 ±0.019	6.583 ±0.520	0.152 ±0.002	0.190 ±0.017	0.185 ±0.010	0.294 ±0.028	0.164 ±0.004	1.170 ±0.012
B		0.086 ±0.007	1.186 ±0.056	1.190 ±0.096	1.237 ±0.079	0.426 ±0.025	6.605 ±0.812	0.556 ±0.014	0.520 ±0.037	0.558 ±0.117	0.594 ±0.148	0.542 ±0.013	1.215 ±0.062
C			0.159 ±0.006	0.706 ±0.024	0.730 ±0.020	1.020 ±0.017	6.592 ±1.171	1.200 ±0.044	1.198 ±0.120	1.201 ±0.413	1.196 ±0.473	1.200 ±0.041	0.748 ±0.069
D				0.195 ±0.024	0.285 ±0.015	1.018 ±0.019	6.589 ±0.696	1.149 ±0.020	1.150 ±0.054	1.137 ±0.179	1.145 ±0.209	1.150 ±0.018	0.318 ±0.023
E					0.169 ±0.021	1.050 ±0.018	6.608 ±0.629	1.189 ±0.017	1.191 ±0.044	1.182 ±0.146	1.185 ±0.170	1.191 ±0.017	0.284 ±0.026
HRAM						0.543 ±0.023	6.576 ±0.517	0.297 ±0.017	0.301 ±0.017	0.317 ±0.023	0.418 ±0.034	0.308 ±0.017	1.056 ±0.018
OUT							7.571 ±0.582	6.583 ±0.521	6.583 ±0.619	6.592 ±1.299	6.544 ±1.471	6.584 ±0.519	6.595 ±0.581
A HRR								0.129 ±0.002	0.157 ±0.004	0.185 ±0.010	0.294 ±0.029	0.164 ±0.004	1.170 ±0.013
A HRT									0.191 ±0.024	0.186 ±0.024	0.292 ±0.048	0.169 ±0.006	1.171 ±0.033
A1B										0.031 ±0.006	0.290 ±0.131	0.197 ±0.011	1.158 ±0.109
A2											0.204 ±0.102	0.303 ±0.029	1.165 ±0.128
AB												0.176 ±0.007	1.171 ±0.012
CDE													0.331 ±0.032

Table S2. Rarefaction analysis. Pairwise genetic distance matrix based on a rarefied dataset comprising 40 randomly selected haplogroup A samples (34 *H.r. rustica* and 6 *H.r. transitiva*). Pairwise genetic distances are highly consistent with those obtained from the complete dataset (Table 1), indicating that rarefaction preserved the overall genetic diversity and relationships among haplogroups.

Haplogroup	Main SubHaplo	Subhaplo	Samples	Expected	Integer
A1	A1a1	A1a1a	54	5.11	5
		A1a1b	42	3.97	4
		A1a1c	8	0.76	1
		A1a1d	9	0.85	1
		A1a1e	4	0.38	0
	A1a2	A1a2a	5	0.47	1
		A1a2b	6	0.57	1
		A1a2c	11	1.04	1
		A1a2d	15	1.42	1
		A1a2e	187	17.68	17
		A1a2f	23	2.17	2
		A1a2g	5	0.47	0
		A1a2h	4	0.38	0
		A1a2i	2	0.19	0
		A1a2j	9	0.85	1
		A1a2k	4	0.38	0
		A1a2l	8	0.76	1
	A1a3	A1a3a	5	0.47	1
		A1a3b	3	0.28	0
	A1a4	A1a4a	2	0.19	0
		A1a4b	5	0.47	1
		A1a4c	3	0.28	0
	A1b	A1b	5	0.47	1
A2	A2	A2	4	0.38	1

of which:		A1a1	1
	H.r. transitiva	A1a2	3
		A1b	1
		A2	1

Hg	A	A trans	B	C	D	E
A	0.14%	0.16%	0.53%	1.20%	1.20%	1.24%
	0.01%	0.03%	0.03%	0.03%	0.05%	0.04%
A trans		0.20%	0.53%	1.21%	1.20%	1.25%
		0.04%	0.08%	0.08%	0.13%	0.11%
B			0.09%	1.19%	1.19%	1.24%
			0.01%	0.06%	0.10%	0.08%
C				0.16%	0.71%	0.73%
				0.01%	0.02%	0.02%
D					0.20%	0.29%
					0.01%	0.02%
E						0.17%
						0.02%

Table S3. Coalescence age estimates of haplogroups using cytb and whole mtDNA. Bayesian age estimates for barn swallow haplogroups and sub-haplogroups and clade separation ages of all available species in the genus.

EXCEL FILE

Table S4. Haplogroup defining mutation in the two control regions for all major haplogroups.

Haplo	Mutation in CR1	Mutation in CR2	Mutation in CR1 specific to sub-haplogroups	Mutation in CR2 specific to sub-haplogroups
A	15082	16987	---	---
B	15082, 15166, 15271, 15280, 15661, 15760, 15777, 16046, 16061C	16987, 17071, 17080, 17176, 17185, 17566, 17665, 17682	---	17195 (B2)
C	15139, 15271, 15280, 15291, 15685, 15696, 16061C	17044, 17075, 17084, 17176, 17185, 17196, 17433, 17601	15661 (C1)	16953 (C2), 17566 (C1)
D	15082, 15139, 15157, 15170, 15183, 15777, 16061C	16779, 16987, 17044, 17075, 17088, 17682	15048 (D2), 15077 (D1), 15661 (D1a), 15696 (D1), 16062 (D2)	16953 (D2), 16982 (D1), 17062 (D1), 17098 (D2), 17101 (D2), 17566 (D1), 17601 (D1)
E	15048, 15082, 15139, 15157, 15170, 15183, 15777, 16061C	16779, 16953, 16987, 17044, 17075, 17088	15696 (E1)	17062 (E1), 17601 (E1)

Table S5. Samples used in this study

EXCEL
